# Autonomic Function and Cerebral Autoregulation in Children Receiving Extracorporeal Life Support

**DOI:** 10.3390/children13030409

**Published:** 2026-03-16

**Authors:** Carlos Castillo-Pinto, Edward Lake, Kin Vong, Thomas V. Brogan, Mark S. Wainwright

**Affiliations:** 1 Department of Neurology, University of Washington School of Medicine, Seattle, WA 98195, USA; lakeedwa@uw.edu (E.L.); mark.wainwright@seattlechildrens.org (M.S.W.); 2 Division of Pediatric Neurology, Seattle Children’s Hospital, Seattle, WA 98105, USA; kin.vong@seattlechildrens.org; 3 Department of Pediatrics, Division of Critical Care, Seattle Children’s Hospital, Seattle, WA 98105, USA; thomas.brogan@seattlechildrens.org

**Keywords:** heart rate variability, cerebral autoregulation, extracorporeal membrane oxygenation, cerebral oximetry index, autonomic dysfunction

## Abstract

**Highlights:**

**What are the main findings?**

- In children supported with ECMO, heart rate variability and cerebral autoregulation showed weak coupling.
- Both NN skewness and COx were independently associated with neurological outcomes without evidence of interaction.

**What are the implications of the main findings?**

- Autonomic function and cerebrovascular regulation represent distinct physiologic domains that independently contribute prognostic information regarding neurological outcomes.
- These findings support the need for multimodal monitoring approaches and larger multicenter studies incorporating high-resolution data to better characterize neurological risk.

**Abstract:**

Background/Objectives: Heart rate variability (HRV) and cerebral autoregulation (CAR) reflect physiologic processes that may influence neurological injury in children supported with extracorporeal membrane oxygenation (ECMO). Although abnormalities in both have been associated with adverse neurological outcomes, their physiologic relationship during ECMO remains unclear. Methods: This retrospective single-center study evaluated the association between HRV and CAR during the first 24 h of ECMO support and assessed their independent relationships with neurological outcome. Patients with at least two hours of simultaneous HRV and CAR monitoring within 24 h of ECMO initiation were included. HRV metrics were derived from artifact-free NN intervals across time, frequency, and nonlinear domains, while CAR was quantified using the cerebral oximetry index (COx), with impaired CAR defined as COx > 0.3. Associations between HRV indices and COx were examined using Spearman correlations at hourly and 24 h resolutions. Unfavorable outcome was defined as death or a Pediatric Cerebral Performance Category (PCPC) score ≥3 at discharge with deterioration from baseline. Results: Eighty-nine patients met inclusion criteria, and 16% demonstrated impaired CAR. HRV measures were reduced relative to age-adjusted norms in both CAR groups without significant differences between groups. Correlations between HRV indices and COx were consistently weak. Overall, 50% experienced unfavorable neurological outcomes. In adjusted logistic regression models, NN skewness and COx were independently associated with outcome, although only NN skewness remained significant in interaction analyses. Conclusions: HRV and CAR exhibited limited physiological coupling during early ECMO support, while each measure provided independent prognostic information with respect to neurological outcome.

## 1. Introduction

Extracorporeal membrane oxygenation (ECMO) is a mechanical support modality used for patients with respiratory or circulatory failure. Though it is a widely implemented life-saving intervention in pediatric intensive care units, mortality rates remain approximately 50% [1,2]. Survivors experience a high burden of morbidity due to an increased risk of neurological complications, including ischemic and hemorrhagic strokes, and seizures [3,4,5,6]. Early identification of patients at risk for neurologic morbidity during ECMO support may enable targeted monitoring and interventions to mitigate secondary brain injury and improve neurological outcomes. However, existing pediatric prediction models primarily focus on mortality and demonstrate only moderate discriminative accuracy [1,2,3,4,5,6,7], underscoring the need for more precise physiological markers to improve risk stratification in this population.

Heart rate variability (HRV) and cerebral autoregulation (CAR) represent two critical physiological systems that may provide complementary insight into brain vulnerability during ECMO. HRV quantifies subtle fluctuations in the intervals between consecutive heartbeats and serves as a marker of autonomic nervous system (ANS) function, reflecting the balance between sympathetic and parasympathetic activity. HRV metrics are categorized into time-domain, frequency-domain, and non-linear measures, each capturing distinct components of autonomic modulation (Table 1). Abnormal HRV has been associated with adverse outcomes across multiple conditions, including cardiovascular and metabolic disorders [8,9,10,11,12], sepsis [13], and traumatic brain injury (TBI) [13,14,15,16].

Cerebral autoregulation (CAR) is a compensatory mechanism that maintains cerebral homeostasis by regulating cerebral blood flow (CBF) in response to fluctuations in systemic blood pressure [19,20]. Impaired CAR may lead to ischemia or hyperperfusion injury when arterial blood pressure falls outside the limits of autoregulation, resulting in secondary brain injury [21,22,23]. In critically ill children, impaired CAR has been consistently associated with unfavorable neurological outcomes across conditions such as TBI and cardiac arrest and during ECMO support [19,20,24,25,26,27].

Impaired CAR and abnormal HRV may share common pathophysiological pathways [28]. Following acute brain injury (ABI), disruption of the ANS—which plays a central role in cardiovascular, respiratory, thermoregulatory, and neuroendocrine regulation—may contribute to this association. ANS dysfunction is frequently observed after ABI and has been implicated as a key mechanism underlying secondary brain injury [27,29,30,31,32,33]. While a potential association of abnormal HRV and impaired CAR during sepsis has been reported in adults [13], pediatric data on this relationship remain limited.

The primary aim of this study was to examine the correlation between HRV and CAR in children undergoing ECMO. The secondary aim was to evaluate the relationship between these physiological processes and neurological outcomes during pediatric ECMO.

## 2. Materials and Methods

### 2.1. Study Design and Data Collection

This retrospective study included children younger than 18 years admitted to any Intensive Care Unit at Seattle Children’s Hospital between March 2020 and June 2025 and received venoarterial (VA) or venovenous (VV) ECMO support. This study was approved by the Seattle Children’s Hospital Institutional Review Board (IRB #00005387) with a waiver of informed consent.

Clinical and demographic data were extracted from the electronic medical record (EPIC; Verona, WI, USA) using in-house data-extraction scripts, while outcome data were obtained by individual chart review. An unfavorable outcome was defined as a PCPC score at hospital discharge ≥ 3 with ≥1 change from baseline. CAR, HRV, and hemodynamic data were derived from simultaneous electrocardiography, near-infrared spectroscopy, and invasive arterial blood pressure recordings obtained during routine clinical care.

Patients were included if at least 2 h of overlapping CAR and HRV data were available within the first 24 h following ECMO initiation. ECMO runs were eligible for inclusion if COx values and all HRV metrics required for analysis were available within this 24 h period. When multiple ECMO runs met these criteria for a given patient, only the earliest ECMO episode was retained.

### 2.2. Cerebral Autoregulation

CAR was quantified using the cerebral oximetry index (COx), defined as the moving Pearson correlation coefficient between the mean arterial blood pressure (MAP) and NIRS-derived cerebral oxygen saturation (StO_2_) [34,35,36]. Artifacts were removed from both signals using automated filters, with outliers defined as values exceeding 3 standard deviations from the mean. After artifact removal, a 5 min moving average was applied to both signals, which were then downsampled into 5 min windows to compute rolling correlations. Impaired CAR was defined as COx ≥ 0.3 [37,38,39].

### 2.3. Heart Rate Variability

ECG signals were acquired from bedside Philips monitors (Philips Healthcare, Andover, MA, USA) and exported for offline analysis. HRV analysis was performed using the PhysioNet Cardiovascular Signal Toolbox [40]. R-peak detection, signal quality assessment, artifact handling, and HRV metric computation were conducted using the toolbox’s automated processing pipeline with default parameter settings. Built-in QRS detection algorithms and signal quality indices were applied to generate beat-to-beat (NN) interval time series; no manual editing or visual confirmation of R-peak detection was performed. Time-domain, frequency-domain, and nonlinear HRV metrics (Table 1) were calculated from the cleaned interval series according to the standardized algorithms implemented within the toolbox.

### 2.4. Statistical Analysis

The resulting COx and HRV metrics were averaged over the 24 h window following ECMO initiation, and for higher-resolution analyses, these metrics were additionally averaged into hourly bins within the same 24 h period. Continuous variables were summarized as median interquartile range (IQR) and categorical variables as counts (percentages). Patients were grouped by CAR status within the 24 h window following ECMO initiation. Comparisons between groups were performed using the Wilcoxon rank-sum test for continuous variables and Fisher’s exact test for categorical variables. All statistical tests were two-sided, and *p* < 0.05 was considered to indicate significance.

Associations between HRV metrics and COx were assessed using Spearman rank correlation coefficients (ρ). Subgroup analyses compared VA and VV ECMO using Fisher’s z-transformation to test differences in correlation strength. For patients with high-resolution (1 h) data, within-patient Spearman correlations (ρ) were calculated between hourly HRV and COx values. Analyses incorporating age-adjusted HRV Z-scores were based on published normative data for healthy pediatric populations [41,42]. Statistical analyses were performed using R version 4.4.0 (R Foundation for Statistical Computing, Vienna, Austria).

## 3. Results

### 3.1. Clinical Characteristics

**Figure 1:**
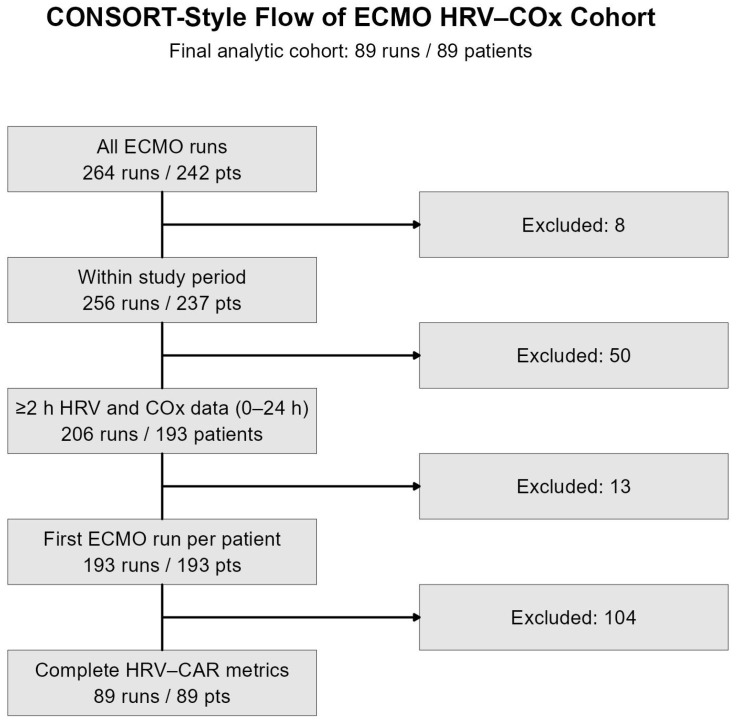
Patient selection flowchart.

After applying inclusion and exclusion criteria, 89 patients had at least 2 h of simultaneous COx data and complete HRV metrics available for analysis (Figure 1). Table 2 summarizes their demographic and clinical characteristics, comparing patients with preserved and impaired cerebral autoregulation. Fourteen patients (16%) demonstrated impaired CAR. Median age at ECMO initiation was 1.8 months (IQR 0.12–13.8), and 56% were male. The majority received VA ECMO (79%), and 21% underwent ECPR. Distribution across ICU locations differed between groups (*p* = 0.046), with patients exhibiting impaired CAR more frequently located in the Neonatal ICU (50% vs. 32%) and less often in the Cardiac ICU (21% vs. 55%). No significant differences were observed in other demographic characteristics, ECMO indication, cannulation site, or underlying comorbid conditions, including prior neurological disorders.

Physiologic and laboratory values were generally similar between groups, except for arterial pH, which was lower in the impaired CAR group, along with a non-significant trend toward higher lactate levels (median 3.3 vs. 1.9 mmol/L; *p* = 0.069). Median ECMO duration was comparable between groups (≈170 h). Patients with impaired CAR showed a trend toward shorter ICU length of stay (median 18.9 [8.8–37.0] vs. 32.9 [18.5–64.2] days) and higher 30-day mortality (36% vs. 15%), although these differences did not reach statistical significance. PCPC-based outcomes at discharge and 12 months were similar between groups.

### 3.2. Cerebral Autoregulation and HRV

Table 3 summarizes heart rate variability (HRV) metrics by COx status. Across all HRV domains, there were no statistically significant differences in HRV indices between both groups. Median values for time-domain measures such as SDNN (10.4 ms vs. 7.5 ms, *p* = 0.22]) and RMSSD (6.9 ms vs. 5.7 ms, *p* = 0.36) were modestly lower among those with impaired CAR, but these differences did not reach significance. Similarly, frequency-domain measures (ULF, VLF, LF, HF) and nonlinear indices (SD1, SD2, PIP, IALS) showed comparable distributions across groups (*p* > 0.14). When analyzed using age-adjusted Z-scores [38,39], RMSSD, SDNN, and pNN50 values were below zero in both groups, indicating globally reduced HRV relative to age norms. However, Z-scores did not differ significantly between groups after adjustment (*p* > 0.39).

All HRV values shown represent mean values computed over the analyzed pre-ECMO window.

When stratified by ECMO type, HRV–COx associations were generally stronger in VV ECMO than VA ECMO (e.g., pip, ials, pas, and nnmean). Although no comparisons reached statistical significance after Fisher’s z-transformation (Supplementary Table S1), several HRV metrics trended toward positive coupling in VV ECMO, whereas most correlations were near-zero or slightly negative in VA ECMO (Supplementary Figure S1).

**Figure 2:**
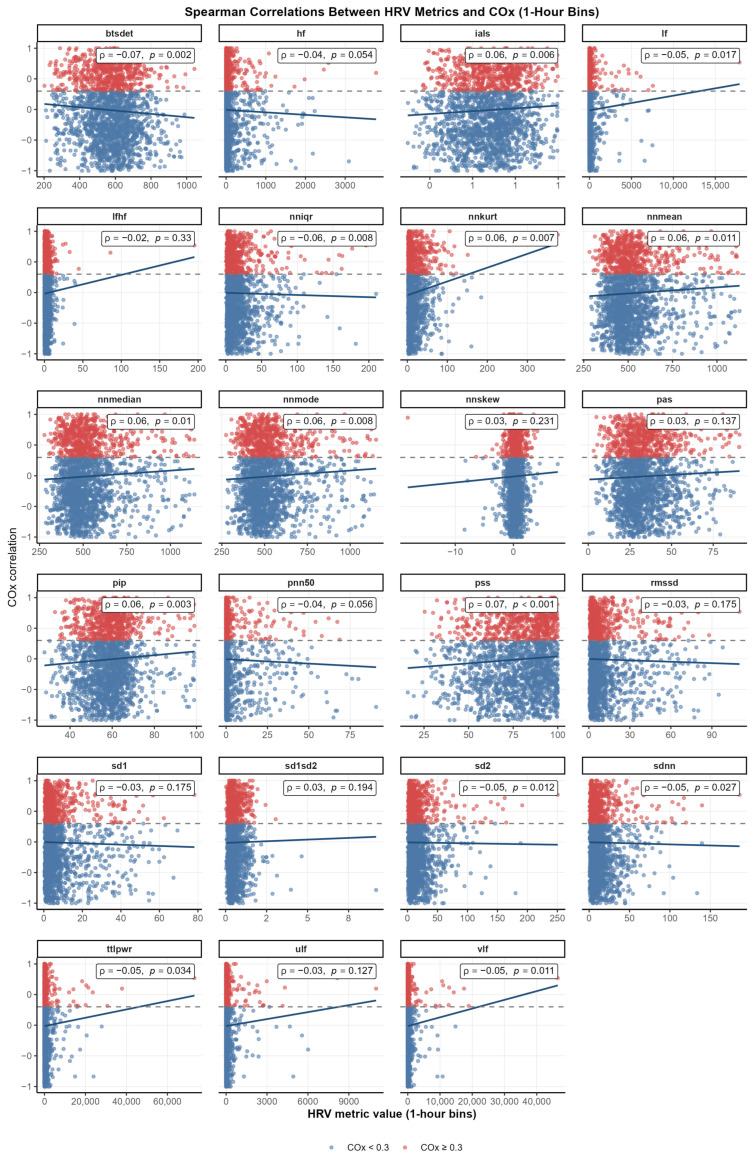
Spearman correlations between HRV metrics and COx by 1 h bins.

To assess whether finer temporal resolution revealed different coupling patterns, HRV–COx correlations were also analyzed using hourly data. Supplementary Figure S2 illustrates the hourly availability of paired HRV and COx measurements across the cohort. Across all HRV metrics, correlations with COx were generally weak, with Spearman’s ρ values ranging from −0.07 to +0.07. Several indices demonstrated statistically significant but small associations with COx. Negative correlations were observed for BTSdet (ρ = −0.07, *p* = 0.002), LF (ρ = −0.05, *p* = 0.017), SDNN (ρ = −0.05, *p* = 0.027), SD2 (ρ = −0.05, *p* = 0.012), and VLF (ρ = −0.05, *p* = 0.011). Weak positive correlations were found for NNmean, NNmedian, and NNmode (all ρ ≈ 0.06, *p* < 0.01), PIP (ρ = 0.06, *p* = 0.003), IALS (ρ = 0.06, *p* = 0.006), NNkurt (ρ = 0.06, *p* = 0.007), and PSS (ρ = 0.07, *p* < 0.001) (Figure 2).

### 3.3. HRV and Outcomes

Unfavorable neurological outcome at discharge occurred in 43 of 86 patients (50%), including death in 31 patients (36%). Patients with unfavorable neurological outcomes had slightly higher COx values compared with those with favorable outcomes (median 0.08 (IQR −0.10 to 0.26) vs. −0.06 (IQR −0.24 to 0.20), *p* = 0.050). In univariate logistic regression analyses, several HRV indices showed significant associations with neurological outcome at hospital discharge. Higher SDNN (OR = 1.04, 95% CI 1.01–1.10, *p* = 0.042), SD2 (OR = 1.04, 95% CI 1.01–1.08, *p* = 0.035), and NN interquartile range (OR = 1.04, 95% CI 1.01–1.09, *p* = 0.028) were associated with greater odds of favorable outcome, while higher NN skewness (OR = 0.38, 95% CI 0.16–0.80, *p* = 0.017) and NN kurtosis (OR = 0.96, 95% CI 0.92–0.99, *p* = 0.019) were associated with lower odds of favorable outcome.

To evaluate whether HRV and COx contributed independent information to outcome prediction, an additive multivariable logistic regression model was constructed that included both NN skewness and continuous COx, adjusting for age, ECMO type, and preexisting conditions. In this model, NN skewness (OR 0.19, 95% CI 0.06–0.48, *p* = 0.001) and COx (OR 0.14, 95% CI 0.02–0.74, *p* = 0.029) were each significantly associated with neurological outcome. A subsequent model incorporating an interaction term (NN skewness × COx) was fitted to evaluate whether the association between HRV and outcome varied across levels of COx. In this interaction model, neither COx (OR 0.24, 95% CI 0.03–1.70, *p* = 0.16) nor the interaction term (OR 0.08, 95% CI 0.00–4.35, *p* = 0.25) achieved statistical significance, whereas NN skewness remained significantly associated with outcome (OR 0.20, 95% CI 0.07–0.50, *p* = 0.001).

## 4. Discussion

In this single-center cohort of pediatric patients supported with VA and VV ECMO, the association between HRV and CAR within the 24 h window immediately following ECMO initiation was weak across all HRV metrics. Correlations between HRV indices and COx were near zero, with only small but statistically significant relationships emerging when analyses were performed at higher temporal resolution. HRV indices were globally reduced relative to age-adjusted reference values and did not differ significantly between patients with preserved versus impaired CAR. In a multivariate model including both NN skewness and continuous COx, each variable demonstrated a statistically significant association with neurological outcome. However, in the interaction model, neither the NN skewness × COx term nor COx remained significant, whereas NN skewness continued to show an independent association with outcome.

Our findings revealed substantially weaker HRV–CAR correlations than those reported in prior studies. Quispe-Cornejo et al. described modest associations between impaired CAR and multiple HRV indices across time, frequency, and nonlinear domains (r ≈ 0.17–0.23) [13]. Following TBI, high-frequency HRV power (0.15–0.40 Hz) demonstrated a strong correlation with the pressure reactivity index (R = 0.63) [43]. The underlying relationship—and potential directionality—between autonomic function, arterial blood pressure, CBF, and intracranial pressure remains poorly characterized.

Several factors may account for the attenuated HRV–CAR coupling observed in our ECMO cohort. First, reduced arterial pulsatility associated with extracorporeal support using centrifugal pump flow attenuates baroreceptor input, leading to decreased baroreflex sensitivity and impaired autonomic regulation [44,45,46]. In addition, administered ICU sedatives—including benzodiazepines, propofol, α2-agonists, opioids, and adjunct agents such as ketamine—are known to reduce sympathetic tone, blunt autonomic variability, and diminish HRV, with effects often related to depth of sedation and overall illness severity [47,48]. Additional contributors may include developmental differences in autonomic maturation [41,42], as well as the possibility that HRV–CAR interactions are nonlinear or highly time-dependent, making them difficult to detect using traditional linear correlation methods.

These relationships emerged only when data were analyzed at higher temporal resolution and were not apparent when using 24 h averages, suggesting that short-term, transient interactions between autonomic tone and cerebrovascular reactivity may occur during ECMO but become diluted over longer timescales. This interpretation is consistent with evidence that short-term HRV measurements are more suitable for clinical assessment, as prolonged recordings are more susceptible to external confounders [49,50]. Because our analysis focused on the first 24 h after cannulation, the observed decoupling may reflect the acute post-cannulation phase, characterized by abrupt hemodynamic shifts, high vasoactive requirements, deep sedation, and reduced arterial pulsatility. Whether this relative independence between HRV and CAR persists during later, more stable phases of ECMO remains uncertain. However, prior studies suggest that both autonomic function and CAR can be altered during ECMO support, influenced by hemodynamic instability, metabolic disturbances, cannulation-related changes in CBF, anticoagulation, and variations in arterial pulsatility [51,52,53,54,55].

Understanding how ANS function contributes to CAR is essential for interpreting HRV–COx relationships in critically ill patients. The ANS plays a central role in regulating CBF through sympathetic and parasympathetic control of vascular tone, and HRV provides a noninvasive measure of ANS activity that may offer indirect insight into neurogenic influences on CAR [13,56]. The cerebral vasculature is richly innervated by autonomic fibers, and fluctuations in sympathetic–parasympathetic balance can influence vasomotor responses to changes in perfusion pressure or metabolic demand [13,57,58]. CAR is also a frequency-dependent process; low-frequency oscillations (0.005–0.05 Hz) are mediated by myogenic, neurogenic, and endothelial mechanisms operating under tonic autonomic control [59,60,61]. Supporting this concept, ganglionic blockade studies have shown that suppression of autonomic input attenuates very-low-frequency blood-pressure oscillations and increases autoregulatory gain, suggesting that autonomic modulation contributes to dynamic cerebrovascular stability [62].

The present analysis extends prior work by evaluating HRV and COx within a unified modeling framework and incorporating both additive and interaction terms to explore their joint relationship with neurological outcome. In the additive model, NN skewness and COx each contributed independently to outcome prediction after adjustment for demographic and clinical factors, suggesting that autonomic variability and CAR may capture complementary dimensions of physiologic instability during ECMO. In contrast, the interaction model did not demonstrate evidence of effect modification between NN skewness and COx, while NN skewness remained independently associated with outcome. Although both HRV alterations and impaired CAR have been associated with worse outcomes in critically ill children, our findings suggest that autonomic and cerebrovascular dysregulation may represent distinct and potentially complementary pathways of neurological risk [26,63,64,65].

This study has several limitations. Only a subset of patients had overlapping HRV and CAR monitoring, and even among those with paired data, hourly measurements were often incomplete. This missingness introduces the potential for selection bias, as patients with greater illness severity or more frequent interruptions in monitoring may have been less likely to contribute complete datasets. Variations in monitoring practices across ICUs may also have led to overrepresentation of certain ICU populations within the analytic cohort. Group sizes were also imbalanced across several comparisons, and some variables had incomplete data availability, which limits subgroup analyses. In addition, multiple HRV–COx correlations were evaluated without formal correction for multiple comparisons. These analyses should therefore be considered exploratory, and the findings—particularly those with small effect sizes—should be interpreted cautiously.

Although age and sex influence HRV, normative reference values for many HRV indices in neonates and young infants remain limited; developmental differences may therefore have contributed to variability in HRV measures given the high proportion of neonates in the sample. The applicability of the COx ≥ 0.3 threshold in neonates should be considered. This cutoff is most commonly used to define impaired CAR in neonatal populations, particularly after cardiac surgery, and is supported by experimental validation studies demonstrating reasonable sensitivity for detecting the lower limit of autoregulation [66,67,68,69,70]. However, some studies have used slightly higher thresholds (e.g., 0.4), reflecting differences in methodology and patient populations [71]. Therefore, this cutoff should be interpreted as a commonly used practical definition rather than a definitive physiologic boundary in neonates and infants.

As an exploratory study, no a priori power calculation was performed, and the analysis may have been underpowered to detect associations or interaction effects. Sedation exposure was evaluated as cumulative burden rather than by specific agents, despite known differences in their effects on HRV and CAR [72,73,74,75,76,77,78]. This was a single-center cohort, which limits generalizability. Finally, the observational design precludes causal inference, and unmeasured confounding cannot be excluded.

## 5. Conclusions

In this cohort of children supported with ECMO, HRV and CAR showed only weak coupling, and their contributions to neurological outcome appeared to be independent rather than interactive. Both NN skewness and COx were associated with outcome in additive modeling, but no interaction between HRV and CAR status was identified. These findings suggest that these processes may reflect distinct physiologic domains relevant to neurological risk during ECMO. Further multicenter studies with more complete high-resolution monitoring data are needed to clarify the temporal and mechanistic relationships between autonomic function, cerebral autoregulation, and neurological outcomes in this population.

## Figures and Tables

**Table 1:** Heart Rate Variability (HRV) Metrics and Definitions.

Abbreviation	Definition/Description	Unit
—Time-domain metrics—		
NNmean	Mean NN interval	ms
NNmedian	Median NN interval	ms
NNmode	Mode NN interval	ms
NNskew	Skewness of NN intervals (distribution asymmetry)	—
NNkurt	Kurtosis of NN intervals (distribution peakedness)	—
NNIQR	Interquartile range of NN intervals	ms
SDNN	Standard deviation of all NN intervals (overall HRV)	ms
RMSSD	Root mean square of successive NN differences (short-term HRV)	ms
pNN50	Percentage of NN intervals differing by >50 ms	%
—Frequency-domain metrics—		
ULF	Power in ultra-low-frequency band (<0.003 Hz)	ms^2^
VLF	Power in very-low-frequency band (0.003–0.04 Hz)	ms^2^
LF	Power in low-frequency band (0.04–0.15 Hz)	ms^2^
HF	Power in high-frequency band (0.15–0.4 Hz)	ms^2^
LF/HF	Ratio of low- to high-frequency power	—
Total Power	Total spectral power (<0.4 Hz)	ms^2^
—Nonlinear (Poincaré) metrics—		
SD1	Short-term HRV (Poincaré SD1)	ms
SD2	Long-term HRV (Poincaré SD2)	ms
SD1/SD2	Ratio of SD1 to SD2 (balance of short- and long-term HRV)	—

Abbreviations and definitions adapted from Task Force of the ESC/NASPE and Bauer et al. [17,18].

**Table 2:** Clinical and Physiological Characteristics by COx Status.

Clinical Characteristic	N	COx Preserved (N = 75) ^1^	COx Impaired (N = 14) ^1^	All (N = 89) ^1^	*p*-Value ^2^
**Age at ECMO (months)**	89	1.80 [0.12, 12.72]	0.12 [0.00, 83.28]	1.80 [0.12, 13.80]	0.5
**Sex**	89				0.6
Male		41 (55%)	9 (64%)	50 (56%)	
Female		34 (45%)	5 (36%)	39 (44%)	
**Indication**	89				>0.9
Respiratory		21 (28%)	4 (29%)	25 (28%)	
Hemodynamic		54 (72%)	10 (71%)	64 (72%)	
**ECPR performed**	89	15 (20%)	4 (29%)	19 (21%)	0.5
**Type (VV/VA)**	89				>0.9
VA		59 (79%)	11 (79%)	70 (79%)	
VV		16 (21%)	3 (21%)	19 (21%)	
**Cannulation site**	52				>0.9
Peripheral		30 (73%)	8 (73%)	38 (73%)	
Central		11 (27%)	3 (27%)	14 (27%)	
**ICU location**	89				0.046
Cardiac ICU		41 (55%)	3 (21%)	44 (49%)	
Neonatal ICU		24 (32%)	7 (50%)	31 (35%)	
Pediatric ICU		10 (13%)	4 (29%)	14 (16%)	
**Race/Ethnicity**	89				0.4
2 or more races		2 (2.7%)	0 (0%)	2 (2.2%)	
American Indian or Alaska Native		6 (8.0%)	1 (7.1%)	7 (7.9%)	
Asian		7 (9.3%)	0 (0%)	7 (7.9%)	
Black or African American		4 (5.3%)	1 (7.1%)	5 (5.6%)	
Hispanic		12 (16%)	5 (36%)	17 (19%)	
Native Hawaiian or Other Pacific Islander		1 (1.3%)	0 (0%)	1 (1.1%)	
Non-Hispanic White		24 (32%)	6 (43%)	30 (34%)	
Other/Unknown		19 (25%)	1 (7.1%)	20 (22%)	
**ECMO run duration (hours)**	89	160.25 [92.42, 392.58]	202.82 [108.43, 406.00]	170.00 [93.00, 392.58]	0.7
**Number of preexisting conditions**	89				>0.9
0		7 (9.3%)	1 (7.1%)	8 (9.0%)	
1		14 (19%)	3 (21%)	17 (19%)	
>2		54 (72%)	10 (71%)	64 (72%)	
**Number of prior neurologic diagnoses**	89				0.4
0		63 (84%)	11 (79%)	74 (83%)	
1		11 (15%)	2 (14%)	13 (15%)	
>2		1 (1.3%)	1 (7.1%)	2 (2.2%)	
**ADHD diagnosis**	89	0 (0%)	1 (7.1%)	1 (1.1%)	0.2
**Autism spectrum disorder**	89	1 (1.3%)	1 (7.1%)	2 (2.2%)	0.3
**Cerebral palsy**	89	1 (1.3%)	1 (7.1%)	2 (2.2%)	0.3
**Developmental delay/** **learning disability**	89	5 (6.7%)	1 (7.1%)	6 (6.7%)	>0.9
**Epilepsy/seizure disorder**	89	1 (1.3%)	1 (7.1%)	2 (2.2%)	0.3
**History of febrile/provoked seizures**	89	2 (2.7%)	0 (0%)	2 (2.2%)	>0.9
**Prior metabolic injury**	89	4 (5.3%)	1 (7.1%)	5 (5.6%)	0.6
**Lactic acid (mmol/L)**	89	1.90 [1.20, 4.30]	3.30 [2.50, 8.60]	2.40 [1.30, 4.30]	0.069
**Hemoglobin (g/dL)**	89	11.30 [10.30, 12.20]	11.70 [11.10, 13.00]	11.30 [10.40, 12.50]	0.3
**pH category**	89				0.034
Severe acidosis (<7.10)		4 (5.3%)	2 (14%)	6 (6.7%)	
Moderate acidosis (7.10–7.24)		5 (6.7%)	1 (7.1%)	6 (6.7%)	
Mild acidosis (7.25–7.34)		28 (37%)	9 (64%)	37 (42%)	
Normal (≥7.35)		38 (51%)	2 (14%)	40 (45%)	
**Arterial pCO_2_ (mmHg)**	89	43.90 [39.50, 48.00]	44.10 [37.30, 47.80]	43.90 [39.50, 47.80]	0.7
**Arterial pO_2_ (mmHg)**	89	124.40 [107.50, 146.80]	131.35 [98.60, 159.50]	124.90 [106.60, 146.80]	>0.9
**End-tidal CO_2_ (mmHg)**	83	24.44 [16.19, 31.22]	25.42 [21.15, 31.57]	24.61 [16.80, 31.32]	0.5
**Rectal temperature (°F)**	54	97.71 [96.54, 98.18]	97.39 [97.08, 97.88]	97.62 [96.54, 98.18]	0.6
**Modified PEWS**	33	11.48 [9.26, 13.59]	13.37 [12.82, 13.92]	12.03 [9.40, 13.76]	0.2
**Number of vasopressor infusions**	89				>0.9
0		22 (29%)	3 (21%)	25 (28%)	
1		27 (36%)	6 (43%)	33 (37%)	
2		18 (24%)	4 (29%)	22 (25%)	
3		8 (11%)	1 (7.1%)	9 (10%)	
**Number of sedative/analgesic infusions**	89				0.7
0		16 (21%)	3 (21%)	19 (21%)	
1		19 (25%)	2 (14%)	21 (24%)	
2		27 (36%)	8 (57%)	35 (39%)	
3		11 (15%)	1 (7.1%)	12 (13%)	
4		2 (2.7%)	0 (0%)	2 (2.2%)	
**Cerebral oxygenation index (COx)**	89	−0.04 [−0.22, 0.13]	0.48 [0.38, 0.64]	0.01 [−0.17, 0.21]	<0.001
**PCPC at admission**	89				0.091
1		63 (84%)	11 (79%)	74 (83%)	
2		5 (6.7%)	2 (14%)	7 (7.9%)	
3		7 (9.3%)	0 (0%)	7 (7.9%)	
4		0 (0%)	1 (7.1%)	1 (1.1%)	
**PCPC at discharge**	86				0.4
1		11 (15%)	1 (7.1%)	12 (14%)	
2		20 (28%)	3 (21%)	23 (27%)	
3		16 (22%)	2 (14%)	18 (21%)	
4		1 (1.4%)	1 (7.1%)	2 (2.3%)	
6		24 (33%)	7 (50%)	31 (36%)	
**PCPC at 12 months**	66				0.9
1		13 (24%)	2 (17%)	15 (23%)	
2		7 (13%)	2 (17%)	9 (14%)	
3		9 (17%)	1 (8.3%)	10 (15%)	
4		1 (1.9%)	0 (0%)	1 (1.5%)	
6		24 (44%)	7 (58%)	31 (47%)	
**ICU length of stay (days)**	89	32.90 [18.47, 64.20]	18.93 [8.78, 37.04]	30.58 [16.18, 58.56]	0.12
**Total hospital stay (days)**	86	56.52 [27.00, 120.50]	23.00 [14.00, 68.00]	49.00 [26.00, 107.96]	0.069
**30-day mortality**	86	11 (15%)	5 (36%)	16 (19%)	0.13
**Mortality at discharge**	86	24 (33%)	7 (50%)	31 (36%)	0.2
**Diffuse edema**	51	2 (4.7%)	2 (25%)	4 (7.8%)	0.11
**Diffuse ischemia**	51	0 (0%)	1 (13%)	1 (2.0%)	0.2
**Focal ischemia**	51	1 (2.3%)	2 (25%)	3 (5.9%)	0.061
**Hemorrhage (extra-axial)**	51	5 (12%)	1 (13%)	6 (12%)	>0.9
**Hemorrhage (intraparenchymal)**	51	1 (2.3%)	1 (13%)	2 (3.9%)	0.3
**IVH (severe)**	51	0 (0%)	0 (0%)	0 (0%)	>0.9
**Any abnormal imaging**	51	9 (21%)	4 (50%)	13 (25%)	0.2

^1^ Median [Q1, Q3]; n (%); ^2^ Wilcoxon rank sum test; Fisher’s exact test; Wilcoxon rank sum exact test.

**Table 3:** Heart Rate Variability (HRV) Metrics by Cox Status.

HRV Metric	N	COx Preserved (N = 75) ^1^	COx Impaired (N = 14) ^1^	All (N = 89) ^1^	*p*-Value ^2^
Mean NN (ms)	89	497.6 [426.2, 561.3]	487.7 [446.6, 559.4]	492.0 [433.8, 559.4]	0.8
Median NN (ms)	89	496.7 [425.7, 560.9]	487.7 [446.2, 559.0]	491.8 [433.7, 559.0]	0.8
Mode NN (ms)	89	495.5 [424.7, 559.4]	487.9 [445.7, 558.2]	491.6 [433.3, 558.2]	0.8
NN Skewness	89	0.2 [−0.1, 0.6]	0.3 [0.1, 0.5]	0.2 [−0.1, 0.6]	0.3
NN Kurtosis	89	12.5 [7.0, 21.2]	16.8 [10.8, 33.0]	12.6 [7.1, 21.6]	0.3
NN IQR (ms)	89	10.4 [7.0, 17.8]	8.3 [4.0, 10.4]	10.2 [6.8, 17.3]	0.14
SDNN (ms)	89	10.4 [6.9, 14.5]	7.5 [4.1, 11.7]	9.9 [6.3, 14.1]	0.2
RMSSD (ms)	89	6.9 [3.4, 12.3]	5.7 [2.8, 7.6]	6.0 [3.2, 12.1]	0.4
pNN50 (%)	89	0.3 [0.1, 2.3]	0.3 [0.2, 0.5]	0.3 [0.1, 1.6]	0.8
Detrended fluctuation (BTS)	89	571.2 [499.6, 677.1]	591.7 [526.4, 604.0]	577.4 [513.0, 660.5]	0.8
ULF Power	89	34.6 [11.3, 68.5]	19.1 [6.5, 45.8]	31.1 [11.1, 64.4]	0.4
VLF Power	89	66.4 [19.6, 163.8]	45.8 [18.8, 126.9]	58.7 [19.6, 161.1]	0.5
LF Power	89	23.1 [8.6, 83.7]	18.6 [8.4, 63.9]	22.4 [8.6, 73.9]	0.6
HF Power	89	28.8 [8.5, 85.4]	26.1 [9.0, 36.4]	26.6 [9.0, 78.5]	0.6
LF/HF Ratio	89	2.0 [0.9, 4.0]	2.0 [1.5, 2.4]	2.0 [1.0, 3.6]	>0.9
Total Power	89	177.0 [85.8, 357.8]	121.0 [45.9, 403.9]	167.9 [75.6, 357.8]	0.3
SD1 (ms)	89	4.9 [2.4, 8.7]	4.0 [2.0, 5.3]	4.3 [2.3, 8.6]	0.4
SD2 (ms)	89	12.1 [7.9, 18.6]	9.5 [5.1, 16.0]	11.4 [7.8, 18.2]	0.3
SD1/SD2 Ratio	89	0.4 [0.3, 0.7]	0.5 [0.4, 0.6]	0.4 [0.3, 0.7]	0.7

^1^ Median [Q1, Q3]; ^2^ Wilcoxon rank sum test.

## Data Availability

The datasets presented in this article are not readily available because the data are part of an ongoing study. Requests to access the datasets should be directed to carlos.castillopinto@seattlechildrens.org.

## Supplementary Materials

The following supporting information can be downloaded at: https://www.mdpi.com/article/10.3390/children13030409/s1, Table S1: Spearman Correlation (ρ) Between HRV Metrics and Cox; Figure S1. Correlation data between HRV metrics and Cox; Figure S2. Availability by hour of patients with both HRV and COx data.

## Abbreviations

The following abbreviations are used in this manuscript:

HRV	Heart Rate Variability
CAR	Cerebral Autoregulation
ECMO	Extracorporeal Membrane Oxygenation
NN	Normal-to-Normal interval
COx	Cerebral Oximetry Index
PCPC	Pediatric Cerebral Performance Category
ANS	Autonomic Nervous System
TBI	Traumatic Brain Injury
CBF	Cerebral Blood Flow
ABI	Acute Brain Injury
VA	Venoarterial (extracorporeal membrane oxygenation)
VV	Venovenous (extracorporeal membrane oxygenation)
MAP	Mean Arterial Pressure
NIRS	Near-infrared Spectroscopy
StO_2_	Cerebral Oxygen Saturation
