# Supplementary material for: Autonomic Function and Cerebral Autoregulation in Children Receiving Extracorporeal Life Support
Source: Children (Basel). 2026 Mar 16;13(3):409. doi: 10.3390/children13030409 (PMC13025630; doi:10.3390/children13030409)
Supplement: Supplementary file 1 [file children-13-00409-s001.zip › children-4175936-supplementary.pdf]

Supplementary Table S1: Spearman Correlation (ϱ) Between HRV Metrics and COx
Comparison Between VA and VV ECMO

HRV Metric	ϱ (VA, n=70)	ϱ (VV, n=19)	p (VA vs VV)
nnmean	-0.01	0.21	0.436
nnmedian	-0.01	0.21	0.435
nnmode	-0.01	0.21	0.438
nnskew	0.12	-0.04	0.567
nnkurt	0.14	0.02	0.663
nniqr	-0.14	-0.12	0.959
sdnn	-0.15	-0.05	0.717
rmssd	-0.14	0.04	0.508
pnn50	-0.13	0.06	0.489
btsdet	0.02	-0.22	0.394
ulf	-0.13	0.13	0.345
vlf	-0.14	0.08	0.435
lf	-0.15	0.00	0.588
hf	-0.16	0.14	0.279
lfhf	-0.07	-0.05	0.940
ttlpwr	-0.16	0.05	0.442
sd1	-0.14	0.04	0.508
sd2	-0.14	-0.04	0.727
sd1sd2	0.04	0.13	0.766
pip	0.07	0.42	0.174
ials	0.11	0.34	0.377
pss	0.14	0.32	0.489
pas	-0.01	0.28	0.282

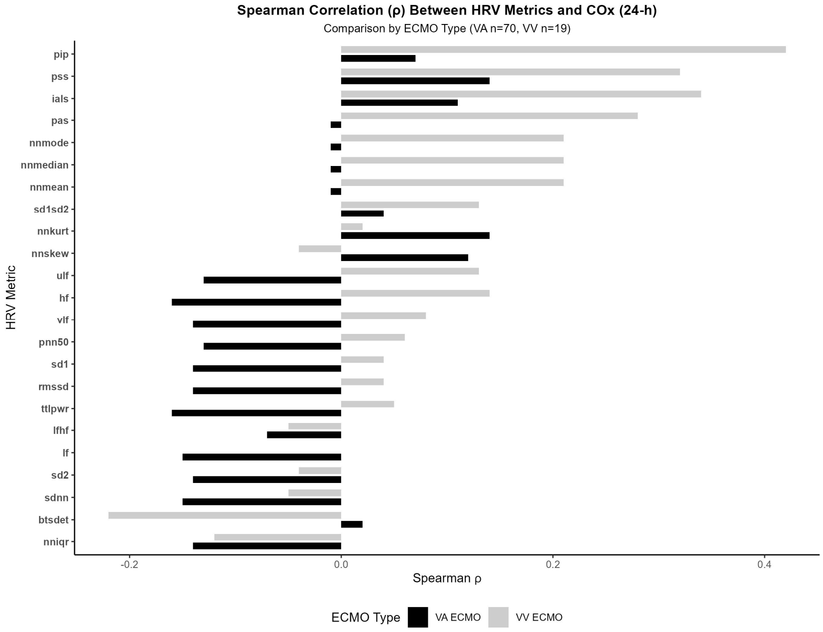

Supplementary Figure S1. Correlation data between HRV metrics and COx.

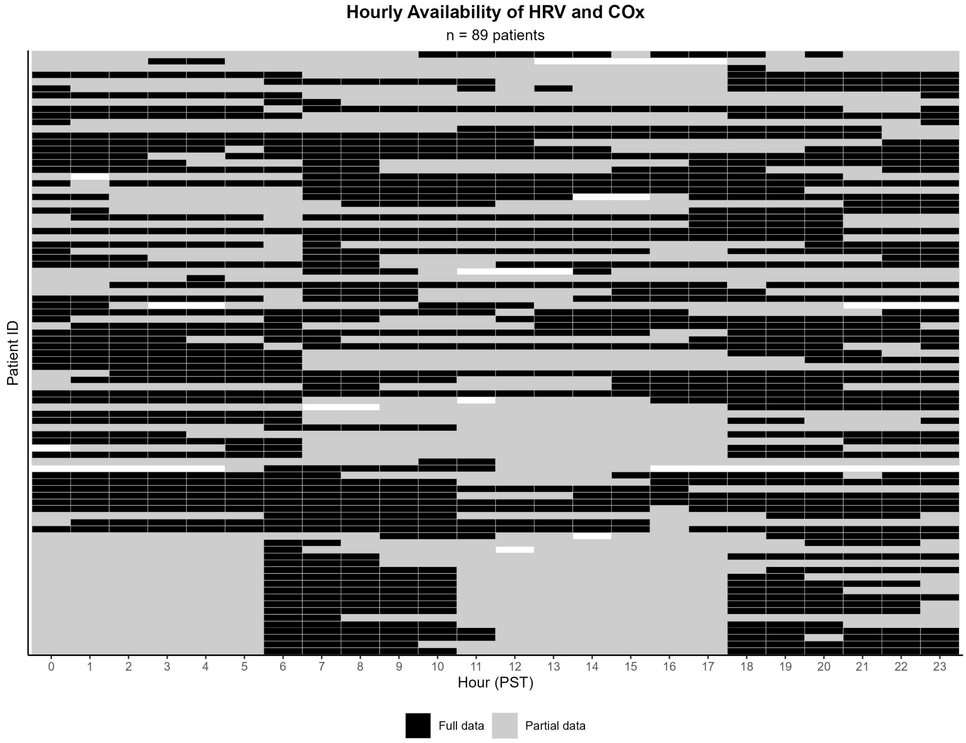

Supplementary Figure S2. Availability by hour of patients with both HRV and COx data.
